# Dielectric characterization of *Plasmodium falciparum*-infected red blood cells using microfluidic impedance cytometry

**DOI:** 10.1098/rsif.2018.0416

**Published:** 2018-10-17

**Authors:** C. Honrado, L. Ciuffreda, D. Spencer, L. Ranford-Cartwright, H. Morgan

**Affiliations:** 1 Faculty of Physical Sciences and Engineering, Institute for Life Sciences, University of Southampton, Southampton, UK; 2 Institute of Infection, Immunity and Inflammation, Animal Health and Comparative Medicine, University of Glasgow, Glasgow, UK; 3 Institute of Biodiversity, Animal Health and Comparative Medicine, University of Glasgow, Glasgow, UK

**Keywords:** human malaria, *Plasmodium falciparum*, impedance cytometry, dielectric characterization, microfluidics

## Abstract

Although malaria is the world's most life-threatening parasitic disease, there is no clear understanding of how certain biophysical properties of infected cells change during the malaria infection cycle. In this article, we use microfluidic impedance cytometry to measure the dielectric properties of *Plasmodium falciparum*-infected red blood cells (*i-*RBCs) at specific time points during the infection cycle. Individual parasites were identified within *i-*RBCs using green fluorescent protein (GFP) emission. The dielectric properties of cell sub-populations were determined using the multi-shell model. Analysis showed that the membrane capacitance and cytoplasmic conductivity of *i-*RBCs increased along the infection time course, due to membrane alterations caused by parasite infection. The volume ratio occupied by the parasite was estimated to vary from less than 10% at earlier stages, to approximately 90% at later stages. This knowledge could be used to develop new label-free cell sorting techniques for sample pre-enrichment, improving diagnosis.

## 1. Introduction

Malaria is undeniably one of the most serious health problems in the world today and is a social and economic burden to many developing countries where the disease is endemic. Estimates of more than 200 million new cases and a death toll of approximately 445 000 for 2016 alone put malaria as the world's most predominant parasitic disease [1]. Five species of *Plasmodium* parasite are known to cause human malaria: *P. falciparum, P. malariae, P. ovale, P. vivax* and the simian parasite *P. knowlesi.* Of these, *P. falciparum* is responsible for the most serious symptomatology and the highest number of deaths, mainly in sub-Saharan Africa.

**Figure 1.**
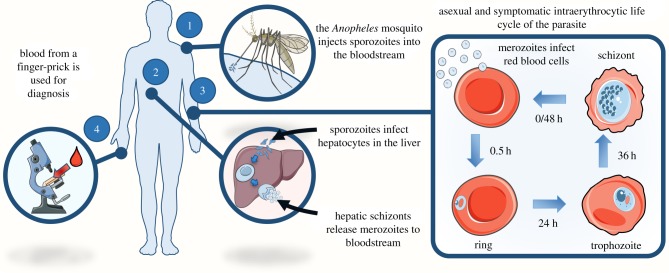
Representation of the *P. falciparum* life cycle in the human host and the common diagnostic method. This includes: 1 – injection of sporozoites into the host by an *Anopheles* mosquito; 2 – parasite invasion of hepatocytes, and consequent production and release of merozoites into the bloodstream; 3 – invasion of RBCs, proceeded by the intraerythrocytic life cycle of the parasite; 4 – diagnosis using microscopy, the current ‘gold-standard’ method, to detect *i-*RBCs in a blood sample.

*Plasmodium* parasites have a complex life cycle, which involves multiple stages in the mosquito vector and human host (figure 1). The infection is established when an infected mosquito of the genus *Anopheles* bites a human host and injects parasites in the sporozoite form into the bloodstream. The parasites reach the liver and invade the hepatocytes, developing into the hepatic schizont, which produces thousands of daughter cells, called merozoites, which are released into the bloodstream. These invade the red blood cells (RBCs). During the intraerythrocytic period, the parasite matures progressively into ring, trophozoite and schizont stages from which newly formed merozoites are released, ready to invade new RBCs and start a new cycle [2].

The intraerythrocytic stage is responsible for the symptomatology of malaria and has a different time length depending on the *Plasmodium* species (24 h for *P. knowlesi*, 48 h for *P. falciparum, P. ovale, P. vivax*, 72 h for *P. malariae).* Throughout the development of the parasite within the host RBC, the host cell is increasingly modified. Although parasite invasion and growth do not cause a significant change in the volume of the infected cell [3], the host cell membrane is profoundly affected. During the invasion process, the malaria parasite primes the RBC surface through binding antigens, altering the biophysical properties of the host cell membrane by reducing the bending modulus and thus lowering the energy barrier restraining the invasion [4]. Furthermore, parasite infection alters the membrane lipid composition and parasite proteins are actively transported to the membrane, creating new permeation pathways (NPPs, also referred to as PSACs, for *Plasmodium* surface anion channels) for metabolite transportation, and altering the cytoskeleton underlying the cell membrane [5–12]. An additional complication in infections due to *P. falciparum* is a phenomenon called ‘sequestration’ [13], where the late-stage parasitized cells adhere to the endothelium of blood capillaries and evade splenic removal of these stiffer cells [14–16]. As a consequence, only ring-stage parasites are able to circulate in the peripheral blood of an infected patient and therefore, are the only stage that can be detected for diagnosis.

The ‘gold standard’ method currently used for malaria diagnosis in endemic areas is microscopy, which is based on the identification of the parasite in RBCs after Giemsa staining. Microscopy is relatively cheap and does not require sophisticated equipment; however, the need for highly skilled technicians, the presence of artefacts or low parasite levels (less than 50 parasites µl^−1^) can affect diagnostic accuracy [17]. The presence of asymptomatic individuals with very low parasite densities highlights the necessity for new diagnostic tools with high sensitivity, especially for areas aiming at malaria eradication [18]. Microfluidic techniques offer solutions to address the issue of diagnosis in low parasitaemia infections. One possible approach is label-free cell sorting, a method that would allow the concentration of parasitized cells within the blood sample without requiring the use of labels [19,20], i.e. exploiting the inherent properties of the cells; the infected cells present in the enriched sample could then be detected using current diagnostic methods with enhanced sensitivity [21].

It is well known that *P. falciparum* parasites gradually modify the infected RBCs (*i-*RBCs) during the intraerythrocytic stage, affecting their mechanical [16,22,23] and dielectric properties [24,25]; the differences in the biophysical properties of *i-*RBCs and uninfected RBCs (*u-*RBCs) have been exploited for separation. Guo *et al*. [26] recently proposed a deformability-based approach where single cells are deformed during ratchet transportation using tapered constrictions and oscillatory flow [26]. The less deformable *i-*RBCs are sorted out from the main, more deformable population of *u-*RBCs, resulting in enrichment levels between 100× and 2500×. Other groups have also used deformability for the detection with some success [27,28]. Nonetheless, these systems are characterized by very low processing volumes and long processing times, making them impracticable alternatives for diagnosis.

AC electrokinetics is a possible approach for the detection of *P. falciparum* parasite-induced changes in the dielectric properties of the *i-*RBC [24,25,29]. Dielectrophoresis (DEP) was used to isolate and enrich *i-*RBCs by up to a factor of 1000× [25]. The dielectric properties of *i-*RBCs at various life cycle stages were measured but the populations were heterogeneous, making it impossible to determine how the dielectric properties change throughout the life cycle of the parasitized cell. More recently, impedance spectroscopy was used to analyse single *i-*RBCs [29] and small changes in the impedance were measured between infected and uninfected cells. However, the samples contained a mixture of parasites at multiple stages, requiring microscopy for post-measurement identification of the *i-*RBCs. Data were only taken at a single frequency (2 MHz), to measure changes in the cell membrane capacitance [30,31], and very few parasites were analysed (e.g. 120 *u-*RBCs and 42 *i-*RBCs in one experiment). The aim of the work presented here is to measure the dielectric properties of the parasitized cell at specific stages in the intraerythrocytic life cycle and to determine whether a label-free discrimination and sorting system exploiting the dielectric properties of *P. falciparum* parasites could be developed.

Full characterization of *P. falciparum i-*RBCs throughout the intraerythrocytic life cycle was performed using microfluidic impedance cytometry (MIC) coupled to a fluorescence microscope. In MIC, single cells suspended in physiological medium flow through a microchannel at high speed and their impedance properties are measured individually at hundreds of cells per second [32,33]. The analysis region comprises a set of parallel facing microelectrodes that are used to measure a small change in current in the presence of a cell. This impedance signal can be analysed to determine cell dielectric properties [34,35]. The technique has been used to discriminate different cell types such as tumour cells in whole blood [33], protozoan parasites [29,36], stem cells [37,38] and leukocytes [39,40]. In some cases [32,38,39,41], a fluorescence-assisted approach was used, enhancing the detection and discrimination capabilities.

In this paper, fluorescence detection was used to unambiguously identify parasites by their expression of the green fluorescent protein (GFP) within the host cell. Data from both *u-*RBCs and *i-*RBCs were modelled using the multi-shell models (based on Maxwell's mixture theory—MMT) to extract their dielectric properties at specific time points along an infection time course (TC). The results are of particular importance in understanding the changes in the parasitized cell during infection and when these appear over the *P. falciparum* intraerythrocytic life cycle.

## 2. Material and methods

### 2.1. System overview

The impedance cytometer has been described previously [32,33,41]. Briefly, a small glass microfluidic chip was fabricated with integrated parallel facing platinum microelectrodes (200 nm thick and 30 µm wide). SU8 photoresist was used to define the microchannel (detection region of 40 µm by 30 µm) by photolithography. The chip was held in a custom 3D-printed holder providing fluidic and electrical connections. The holder was mounted on a *xyz* stage within a bespoke fluorescence microscope for simultaneous measurements of single cell impedance and fluorescence. Samples were measured at the flow rate of 40 µl min^−^¹. Between measurements, chips were flushed with 1 ml deionized (DI) water and 1 ml phosphate-buffered saline (PBS). Periodic cleaning steps were taken to remove debris from electrode surfaces by flushing with 1 M sodium hydroxide before rinsing with 1 ml DI water.

**Figure 2.**
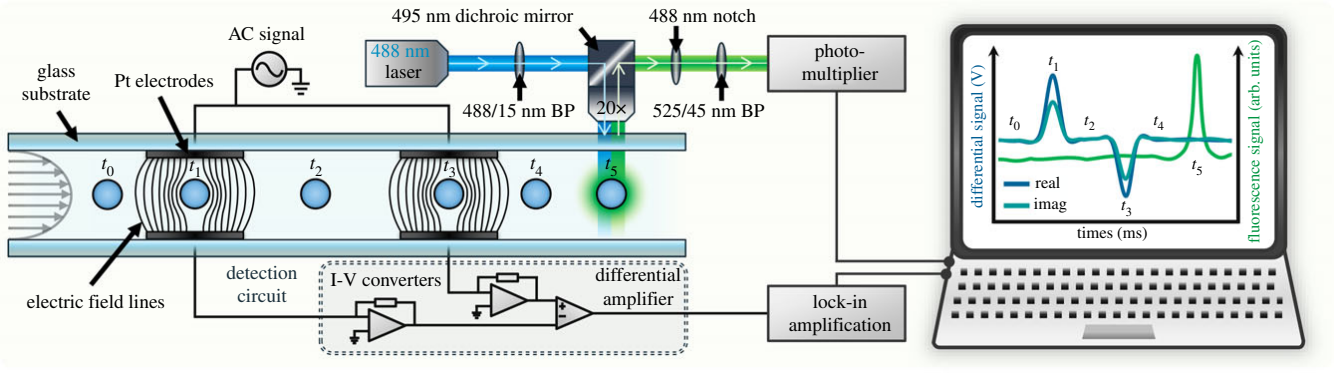
Schematic of the MIC system, showing impedance and fluorescence detection sections. Particles flow through the microchannel, between pairs of electrodes and the fluorescence detection region. The fluorescence from cells is measured simultaneously with impedance, allowing direct correlation of electrical and fluorescent properties of single cells.

A schematic diagram of the system is shown in figure 2. Sinusoidal voltages at two fixed frequencies were applied to both top electrodes. The first (reference) voltage was applied at a frequency of 18.3 MHz, while a second frequency (probe frequency) was varied in steps from 250 kHz and 50 MHz. The current flowing through each electrode was converted to voltage and a differential output signal, measured with a home-made differential amplifier and a lock-in amplifier (HF2FLI, Zurich Instruments), to give the real and imaginary parts of the signal at each frequency. Simultaneously, a 488 nm laser beam (L488P60, Thorlabs) illuminated the detection region. As *i-*RBCs flow through this region, GFP within parasites is excited. The emitted light was captured with a 20× objective lens, passed through a dichroic mirror and filters, and detected with a photomultiplier. Both impedance and fluorescence signals for each cell were therefore captured simultaneously.

### 2.2. Sample preparation

*P. falciparum* parasites genetically modified to express GFP [42] were grown according to standard protocols [43]. For TC experiments, MACS columns [44] were used to purify schizonts from an asynchronous culture. Purified schizonts were then allowed to reinvade fresh RBCs in a shaking incubator for 4 h. At the end of the 4 h incubation, the parasite culture was treated with sorbitol [45] to lyse all schizont stage parasites that had not burst and reinvaded before that time. Only ring-stage parasites survived sorbitol treatment, resulting in a synchronized culture of parasitized RBCs ranging in age from 0 to 4 h post-invasion (hpi) which was used for subsequent measurements. Cultures were grown on, and samples removed at 6, 12, 18, 24, 30, 36, 42 hpi for TC measurements. ‘Time zero’ (*t* = 0) was set at the half point of the invasion window of the sorbitol-treated culture; in this way, parasites at each time point had a time = hpi ± 2 h. A control sample containing only RBCs (control-RBCs (*c-*RBCs)) was kept under the same conditions and was measured in parallel with the parasite culture. Uninfected erythrocytes exposed to culture conditions are denoted *u*-RBCs. Parasitaemia and the stages of the parasites present at each time point were assessed using Giemsa-stained thin blood smears. Prior to measurements, all samples were re-suspended in PBS to a final concentration of 500 cells µl^−1^. Polystyrene beads (7 µm diameter; Sigma-Aldrich, Germany) were also added as reference particles to a final concentration of 100 beads µl^−1^.

### 2.3. Data analysis

Data were processed and analysed using a custom script written in Matlab (R2016a). The signal from reference beads was used to normalize both the fluorescence and impedance data. Scatter plots of infected and uninfected cell populations were drawn with a mean and an elliptical boundary containing 50% of each population (assuming a normal distribution). A covariance matrix was first calculated, identifying the directions in which the 2D data of a single population varied most. The smallest and largest eigen-vector of the matrix gives the direction of data spread, while their respective eigen-values give the spread magnitude. Next, based on each population spread and orientation, a confidence ellipse, containing within 50% of all events for a given population, was plotted. The centre of the ellipse gives the population mean position.

### 2.4. Statistical analysis

Statistical analysis was performed using a custom script in Matlab (R2016a). The statistical significance of the differences in parasitaemia obtained from Giemsa staining/light microscopy- and fluorescence/MIC-based methods was assessed using Pearson's chi-squared test, by comparing the proportions of *i-*RBCs in each RBC populations. The statistical significance of the difference between TC data (real and imaginary parts) was assessed using Student's *t-*test by comparing the mean values of each TC along the 24 probe frequencies. The statistical significance of the differences in the dielectric properties of cells was assessed using Student's *t-*test by comparing the values of different cell populations for single time points. The statistical significance of the differences in dielectric properties of *i-*RBCs was first assessed by an ANOVA test for differences within the time points, followed by Tukey's test, by performing pairwise comparisons between each time point. Results are represented as mean ± s.d. unless otherwise stated.

### 2.5. Optical flow cytometry

Conventional flow cytometric analysis of GFP parasites at the ring and mature stages was carried out using a BD LSRFortessa with a 488 nm laser. *u*-RBCs were measured as control samples. Asynchronous parasite cultures were treated with sorbitol 24 h before the experiment or on the same day as the experiment to produce samples with mature stages and ring stages, respectively. Parasitaemia and the stages of the parasites present immediately before the measurements were assessed using Giemsa-stained thin blood smears. Cytometric data were exported as standard FCS files, and the forward and side scattered light (FSC and SSC) and fluorescence (FITC region) signals were analysed using FlowJo, LLC (V.10).

### 2.6. Data modelling

Data were modelled using a custom script written in Matlab (R2016a). An iterative algorithm generated multiple relaxation curves based on the multi-shell model and MMT [30,31,46–49]. Electronic supplementary material, section B describes how this model was implemented to generate relaxation curves for the real and imaginary parts of impedance using the following parameters: mean values for the semi-axis (*n* = *a*, *b* and *c*) [3]; suspension medium conductivity 1.5 S m^−1^ and permittivity 80; and fixed membrane thickness 5 nm [50] and conductivity ≤10^−8^ S m^−1^). The relaxation curves were normalized against the reference beads (*σ*_beads_ = ≤10^−3^ S m^−1^; *ɛ*_beads_ = 2.5), permitting a direct comparison with the normalized experimental data. The algorithm used a pattern search function to find the local minimum between a measured dataset and the spectrum derived from the model, i.e. which variables generate a relaxation curve that has the minimal difference between data and model. The pattern search function required an initial vector, with starting points for each variable, i.e. for each dielectric property being modelled; as well as two boundary vectors, defining the maximum and minimum values for each variable. The real and imaginary parts were modelled at the same time, with the local minimum difference being calculated considering the differences in both parts. The fitting process ended when the local difference between the data and model was smaller than a predefined tolerance value, with an *R*^2^ value evaluating the goodness of fit.

Each measured dataset comprised mean values of the real and imaginary parts of the impedance signal, along the 24 probe frequencies analysed. The mean values were derived from a population of approximately 3000 to approximately 5000 events/frequency for *c-*RBCs and *u-*RBCs, while for *i-*RBCs the number of events varied according to the parasitaemia in each individual experiment (approx. 150 to approximately 350 events/frequency). For early-stage *i-*RBCs (6–18 hpi), to reduce the complexity of the modelling process and increase the confidence in the results, certain variables were fixed as follows: *ɛ*_cell cytoplasm_ = 60, *ɛ*_par cytoplasm_ = 60, *σ*_par cytoplasm_ = 0.40 S/m [25,51–54].

## 3. Results and discussion

### 3.1. Identification of infected cells

**Figure 3.**
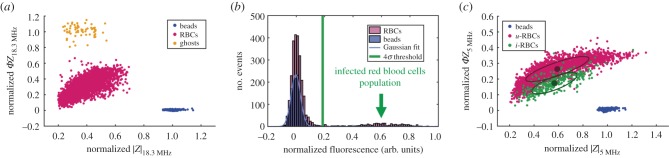
Identification of infected cells using combined impedance and fluorescence data. (*a*) Normalized impedance scatter plot (magnitude |*Z*| versus phase *ΦZ*), at the reference frequency (18.3 MHz), of a culture containing parasite-infected cells at 30 hpi, mixed with reference beads. (*b*) Normalized fluorescence distributions of the same culture at 30 hpi, with reference beads, used for the identification of infected cells. (*c*) Normalized impedance scatter plot (magnitude versus phase), measured at a frequency of 5 MHz for the same culture at 30 hpi, with reference beads, showing discrimination between *u-*RBCs and *i-*RBCs. The ellipse containing 50% of each population together with the mean (filled circle) are also indicated.

The use of parasites genetically modified to express GFP throughout the intraerythrocytic lifecycle [42] ensured *i-*RBCs were identified independently of any changes in dielectric properties. Identification of the *i-*RBCs was done with the protocol summarized in figure 3. Firstly, the impedance scatter plot was generated at a reference frequency (18.3 MHz) (data not shown) and the data normalized against the 7 µm reference polystyrene beads. Figure 3*a* shows normalized impedance scatter plots for a culture containing parasite-infected cells at 30 hpi, mixed with reference beads. It is known that the impedance of the beads depends on their position within the detection region, i.e. the trajectory of the bead as it flows between the electrodes [55]. After correction for this effect [56], the impedance magnitude of reference beads is normally distributed around a mean value (as in figure 3*a*,*c*). In addition to the bead population, two other clusters are seen: one corresponding to all RBCs (both *i-*RBCs and *u-*RBCs), and another corresponding to non-viable RBCs, or ‘ghosts’. These ghosts are usually present in processed blood samples (as in control samples—electronic supplementary material, figure S1a) and will be ignored in the remaining analysis.

Infected cells were uniquely identified by their fluorescence. Conventional flow cytometry confirmed the presence of *i-*RBCs at both early and late stages of infection (electronic supplementary material, figure S2). Figure 3*b* shows a histogram of the fluorescence for beads and all RBCs. A second, brighter population of RBCs was seen in the histogram, corresponding to the *i-*RBCs. This brighter sub-population was not observed in control samples with no exposure to parasites (see electronic supplementary material, figures S1b and S2a,d). To define the boundary between infected or otherwise, the bead fluorescence data were fitted to a Gaussian distribution. The infected cells were gated from uninfected cells by defining a threshold equal to 4× the s.d. (*σ*) away from the mean fluorescence of the beads, giving the scatter plot shown in figure 3*c*. In this way, normalized impedance scatter plots were generated for each probe frequency and each time point during the infection.

### 3.2. Time course experiments

MIC measurements were made throughout the parasite intraerythrocytic cycle every 6 h. The first measurement was performed at 6 hpi, with the last measurement at 42 hpi (control samples (*c*-RBCs) were also measured at the same time points). Three sets of TC experiments were performed. No significant difference in real and imaginary parts was found between the TCs (Student's *t-*test, *N* = 24 frequencies; *p* ≫ 0.05). For each experiment, the parasitaemia, i.e. the percentage of infected cells within the RBC population established from Giemsa-stained blood smears was: TC1 = 10.3 ± 0.96%, TC2 = 8.18 ± 0.87% and TC3 = 5.39 ± 0.71% (mean ± s.e.m. for *N* = 7 time points; 1000 RBCs counted per time point). To assess the system sensitivity, the parasitaemia at individual time points for each TC was calculated using the number of *i-*RBCs identified by fluorescence-coupled MIC (from a total of *N* = 4000 events per time point). Electronic supplementary material, figure S3 compares the parasitaemias calculated by Giemsa staining/light microscopy and fluorescence-coupled MIC; and the mean parasitaemia calculated by fluorescence was: TC1 = 9.77 ± 1.06%, TC2 = 5.64 ± 0.62%, and TC3 = 4.15 ± 0.63% (*N* = 7 time points).

It was observed that at certain time points, the parasitaemia was lower when calculated by fluorescence-coupled MIC than by Giemsa staining/light microscopy (electronic supplementary material, table S1). At early time points (6 hpi), this difference was significant for two of the three TCs (*p* < 0.0001 for TC2 and *p* < 0.01 for TC3; Pearson's chi-squared test for proportions of *i-*RBCs in RBCs populations, *N* = 7 time points). One explanation for the discrepancy could be the presence of younger parasites that had yet not produced sufficient GFP to be detected. GFP expression in the parasite line is under the control of the promoter for the histidine rich protein III (*hrpIII:Pf3D7_1372200*) gene, for which RNA-seq data indicates lower expression in the first 5 hpi [57]. Owing to the synchronization protocol and small differences in the life cycle, parasites at each time point span an invasion window of hpi ± 2 h (see Sample preparation section in Material and methods). Consequently, at the 6 hpi time point, while TC1 parasites could be at a time point greater than or equal to 6 hpi, TC2/3 parasites could be at a *de facto* time point less than 6 hpi (4–6 hpi, when lower GFP expression is expected), which would explain the reduced fluorescence and lower parasitaemia for these TCs.

This phenomenon was also observed by flow cytometry (electronic supplementary material, figure S2b and S2e), where the percentage of *i-*RBCs identified by fluorescence was significantly lower (7.5%) than the one identified by Giemsa staining/light microscopy (9.5%) for early-stage samples (*p* < 0.05; Pearson's chi-squared test), while no significant difference was observed for late-stage parasites (*p* > 0.05). In TC2, the difference for most time points (*p* < 0.05) was probably systematic (e.g. a drift in the laser beam focal point, which would affect the sensitivity of the system, or the presence of debris in the detection region). Nevertheless, parasitaemias calculated by fluorescence-coupled MIC were always close to those determined by microscopy. In the case of control samples containing only *u-*RBCs, very small percentages (0.3 ± 0.1%) of cells were above the threshold fluorescence and classified as ‘*i-*RBCs’ (electronic supplementary material, table S2). Thus, the majority of true *i-*RBCs were detected for every time point, in each TC.

**Figure 4.**
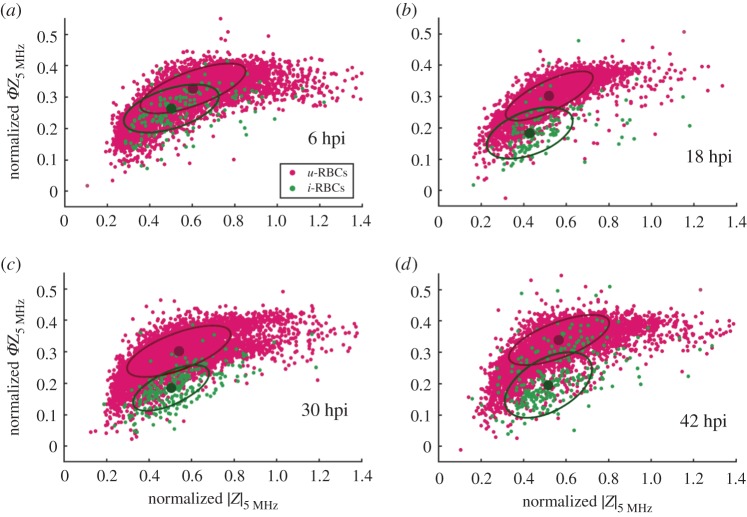
Normalized impedance scatter plots (magnitude |*Z*| versus phase *ΦZ*), at a frequency of 5 MHz for parasite culture samples at (*a*) 6 hpi, (*b*) 18 hpi, (*c*) 30 hpi and (*d*) 42 hpi, showing discrimination between *u-*RBCs and *i-*RBCs. The mean (filled circle) and ellipse containing 50% of each population are also indicated. Data from TC3.

The impedance data for *u-*RBC and *i-*RBC populations at different hpi in one experiment are shown in figure 4 (and electronic supplementary material, figure S4)—data from TC3, with similar behaviour observed in TC1 and TC2. For very early stages of infection (6 hpi; figure 4*a*), there was a high degree of overlap between the two cell populations. As the parasite developed intraerythrocytically, the *i-*RBC population shifted in impedance phase (18 hpi; figure 4*b*), with optimal discrimination at later stages of infection (30 hpi; figure 4*c* and 36 hpi; electronic supplementary material, figure S4f). Interestingly, discrimination decreased at a very late time point (42 hpi; figure 4*d*). At this point in the cycle, some of the late-stage parasites have already burst because each time point has a 2 h window (hpi ± 2 h). These parasites would begin infecting new cells, explaining the increased number of *i-*RBCs events overlapping the *u-*RBCs population. However, this was a small fraction of the infected population and did not affect the dielectric characterization. From the time-dependent data, it appears that there is no specific time window where the parasite can be unambiguously detected. Clear discrimination between uninfected and infected cells would be especially difficult at early stages of infection.

The changes in discrimination were observed at frequencies between 2 and 8 MHz (figure 4 shows observations at 5 MHz). According to dielectric theory, this range of frequencies measures cell membrane properties (in PBS) [30,31] suggesting discrimination based on membrane properties as parasite growth progresses, in accordance with the literature [25]. This trend was not reported in Du *et al*. (2013) where only a single frequency (2 MHz) was used [29]. The authors measured the impedance of *i-*RBCs and *u-*RBCs and found no difference between adjacent stages of *i-*RBCs. These observations are contrary to expected, as the parasite induces changes in the mechanical [16,22,23] and dielectric [24,25] properties of *i-*RBCs membrane as it grows within the host cell.

### 3.3. Impedance data modelling

**Figure 5.**
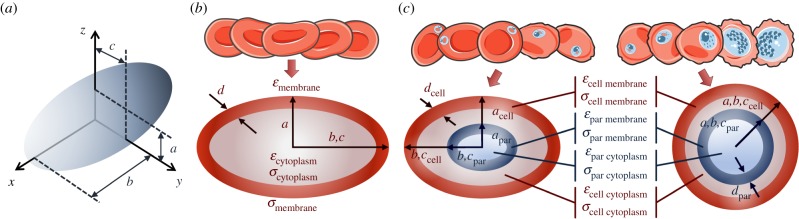
(*a*) Ellipsoidal model, with semi-axes *a*–*c*. (*b*) Oblate spheroid model implemented for *u-*RBCs, with semi-axes *a* < *b* = *c* (based on the ellipsoidal model), membrane thickness *d* and dielectric properties modelled are represented. (*c*) Models implemented for *i-*RBCs: oblate spheroid model for early-stage *i-*RBCs and spherical model for late-stage *i-*RBCs, with semi-axes *a* = *b* = *c* (based on the ellipsoidal model). Membrane thickness of cell (*d*_cell_) and parasite (*d*_par_), and dielectric properties modelled are represented.

While many biological cells can be approximated to a spherical particle, the discoid shape of RBCs requires an alternate ellipsoidal model as shown in figure 5*a* [31,46–48]. This model was used to determine the dielectric properties of the RBC—as described in the Data modeling section of Material and methods, and electronic supplementary material, section B. For the RBC, the model approximates the cell to a single-shell oblate spheroid, with semi-axes *a* < *b* = *c* (figure 5*b*). The model was first used to determine the dielectric properties of the control *c-*RBCs (electronic supplementary material, figure S5 and table S3), with good agreement to the widely accepted values (*R*^2^ = 0.9990 ± 0.0004; *N* = 7 time points) [25,51–54]. The model was then implemented for the *u-*RBCs in the parasite cultures for all time points (*R*^2^ = 0.9993 ± 0.0004; *N* = 7 time points). Estimated dielectric properties of *u-*RBCs were also in agreement with literature values [25,51–54] (electronic supplementary material, figure S6 and table S4), suggesting that the exposure of *u-*RBC to parasites and their secreted metabolites under culture conditions does not significantly alter the dielectric properties of the RBC (no bystander effect [58]).

**Figure 6.**
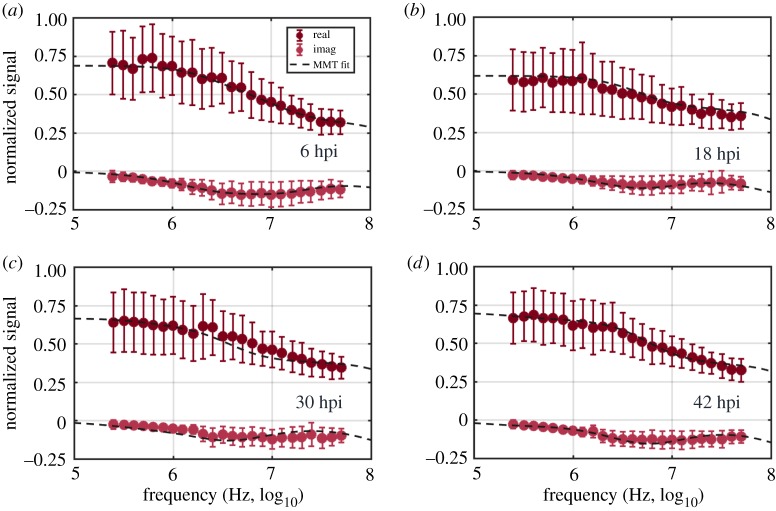
Normalized real and imaginary parts of the average impedance spectrum of *i-*RBCs, across the measured probe frequency spectrum, of *i-*RBC culture samples at (*a*) 6hpi, (*b*) 18 hpi, (*c*) 30 hpi and (*d*) 42 hpi. Double-shell oblate spheroid (*a*,*b*) and spherical (*c*,*d*) models were used to generate each MMT fit. The optimal MMT fits (dashed lines) are plotted on top of individual probe frequency mean values (circles) and s.d. (error bars) for each time point. Data from TC1.

For the *i-*RBCs, the wide variations in size and shape of host cell and parasite throughout the parasite intraerythrocytic life cycle necessitated the use of two different models depending on the stage of infection (figure 5*c*). For early-stage *i-*RBCs (from 6 to 18 hpi), the ellipsoidal model was used. Post-invasion, the parasite flattens into a thin flat or cup-shaped disc ring and does not alter the shape of the host cell until later [3,59,60]. Thus, a double-shell oblate spheroid model was used to model the presence of the parasite within the host cell. The ring-stage parasite changes to a more rounded and irregular trophozoite, and later schizont, form. These changes are accompanied by an increase in parasite volume, changing the host cell to a less discoid shape [3,59,60]. The increased parasite size and shape changes in the host cell render an ellipsoid model inaccurate, so that for late-stage *i-*RBCs (greater than or equal to 24 hpi), the double-shell spherical model was used (with semi-axes *a* = *b* = *c*, and the same initial modelling conditions). This approach gave optimal fits for all time points (*R*^2^ = 0.9972 ± 0.0021; *N* = 7 time points) (figure 6; electronic supplementary material, figure S7 shows all time points for TC1).

The dielectric properties of both host cell and parasite were determined. Tables summarizing the dielectric properties for *c-*RBCs, *u-*RBCs and *i-*RBCs and parasite can be found in the electronic supplementary material, tables S3, S4 and table 1, respectively.

**Table 1.** Dielectric properties of *i*-RBCs and parasite, estimated using MMT modelling, during the parasite intraerythrocytic life cycle (hours post-invasion, hpi). Values are mean estimates (*N* = 3 TCs) with s.d.

	period of intraerythrocytic development of parasite						
properties	6 hpi	12 hpi	18 hpi	24 hpi	30 hpi	36 hpi	42 hpi
*ɛ*_cell membrane_	4.58 ± 0.28	5.38 ± 0.36	6.43 ± 0.50	7.40 ± 0.43	7.99 ± 0.80	8.32 ± 0.45	7.80 ± 1.40
*ɛ*_cell cytoplasm_	60 (fixed value)	60 (fixed value)	60 (fixed value)	66.7 ± 9.4	83.3 ± 4.7	86.7 ± 4.7	86.7 ± 4.7
*ɛ*_par membrane_	3.03 ± 0.40	3.33 ± 0.47	5.83 ± 0.24	5.67 ± 0.47	5.67 ± 1.70	3.50 ± 0.41	4.00 ± 0.82
*ɛ*_par cytoplasm_	60 (fixed value)	60 (fixed value)	60 (fixed value)	76.7 ± 9.4	76.7 ± 4.7	86.7 ± 4.7	86.7 ± 4.7
*σ*_cell cytoplasm_ (S m^−1^)	0.51 ± 0.03	0.52 ± 0.02	0.55 ± 0.04	0.53 ± 0.05	0.74 ± 0.04	0.97 ± 0.07	1.24 ± 0.04
*σ*_par cytoplasm_ (S m^−1^)	0.40 (fixed value)	0.40 (fixed value)	0.40 (fixed value)	0.50 ± 0.09	0.44 ± 0.04	0.47 ± 0.01	0.50 ± 0.03
*C*_cell membrane_ (mF m^−2^)	8.1 ± 0.50	9.1 ± 0.47	11.4 ± 0.88	13.1 ± 0.77	14.1 ± 1.42	14.8 ± 0.83	13.8 ± 2.47

**Figure 7.**
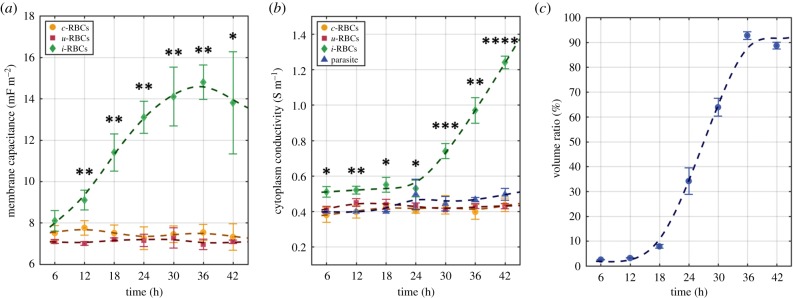
Dielectric properties, estimated using MMT modelling, during the parasite intraerythrocytic life cycle: (*a*) membrane capacitance, (*b*) cytoplasm conductivity and (*c*) volume ratio occupied by parasite within the host cell. Mean values from the three TC experiments (*symbols*) and s.d. (*error bars*) are plotted for each time point. Smoothing splines (dashed lines) are plotted to represent the overall trend for each population. Statistical significance (Student's *t-*test; *p < 0.05, **p < 0.01, ***p < 0.001 and ****p < 0.0001) is represented for *i-*RBCs (*N* = 3) versus *c-*RBCs and *u-*RBCs (*N* = 6) at individual time points.

Figure 7 shows the time-dependent change in the dielectric properties of *c-*RBCs, *u-*RBCs, *i-*RBCs and parasites post-invasion. Statistical analysis showed no significant difference between the dielectric properties of *u-*RBCs and *c-*RBCs (Student's *t-*test, *N* = 7 time points; *p* ≫ 0.05). Consequently, both populations were used for comparison with *i-*RBCs, at different time points (Student's *t-*test, *N* = 7 time points). Statistical analysis showed significant differences in dielectric properties of *i-*RBCs within the TCs (ANOVA test, *N* = 7 time points; *p* ≪ 0.05). Thus, changes in *i-*RBCs during the infection cycle were statistically assessed by comparing host cell membrane capacitance and cytoplasmic conductivity for *i-*RBCs at individual time points (Tukey's test, *N* = 21 pairwise comparisons; electronic supplementary material, tables S5 and S6).

As previously discussed (figure 4), one of the aspects that distinguish uninfected from *i-*RBCs is the alterations seen in the host cell membrane. The *u*-RBCs were found to have a mean membrane capacitance similar to previously published values (*c-*RBCs − *C*_membrane_ = 7.48 ± 0.15 mF m^−2^; *u-*RBCs − *C*_membrane_ = 7.11 ± 0.10 mF m^−2^; *N* = 7 time points) [25,52–54]. By contrast, the membrane capacitance of *i-*RBCs varied during infection (figure 7*a*) and became significantly different to the uninfected cells as early as 12 hpi. The membrane capacitance of *i-*RBCs increased at this time point to a significantly higher level (*C*_cell membrane_ = 9.1 ± 0.47 mF m^−2^) than *u-*RBCs (*p* < 0.01). The membrane capacitance of the *i-*RBCs increased gradually throughout the period of parasite development within the RBC (*p* < 0.01 between 6 and 36 hpi), reaching 14.1 ± 1.42 mF m^−2^ at 30 hpi. Active remodelling of the host cell membrane occurs from the very early stages of parasite growth and is likely to be the reason for this gradual change in membrane capacitance [5–7]. Membrane capacitance is a function of membrane thickness, area and permittivity [31]. Although the surface area of the RBC is not affected at early stages of infection [3], the host cell membrane lipid composition is modified by the parasite during infection [11], which could alter membrane permittivity, and consequently membrane capacitance. Also, after merozoite invasion and establishment as a ring-stage parasite within the RBC, proteins involved in active remodelling of the host cell are produced, transported across the host cell cytoplasm and inserted in the RBC membrane, modifying its composition and possibly capacitance [61]. As the parasite develops further, a channel-like system known as the new permeation pathway (NPP) appears on the host plasma membrane 10–20 hpi [7], and results in an increased ability of parasite-infected erythrocytes to take up nutrients, especially anionic compounds [12]. Furthermore, the accumulation of parasite proteins at the host cell membrane underside causes the appearance of small angular elevations (*knobs*) at later stages, altering the membrane structure and surface area [59,60], which could increase the capacitance.

One of the consequences of the membrane alterations is an increase in ion exchange activity between the intra- and extracellular regions. This effect was observed as the parasite matures, with significant increases in cytoplasmic conductivity (figure 7*b*). The conductivity of *i-*RBCs cytoplasm was already significantly different at 6 hpi (*σ*_cell cytoplasm_ = 0.51 ± 0.03 S m^−1^, *p* < 0.05) to that of *u-*RBCs (*c-*RBCs: *σ*_cytoplasm_ = 0.38 ± 0.04 S m^−1^; and *u-*RBCs:*σ*_cytoplasm_ = 0.41 ± 0.02 S m^−1^). While it is possible that these differences result from the invasion process, with some of the extracellular medium entering the host cell together with the parasite, there was no significant increase in cytoplasmic conductivity during the ring stage of infection suggesting this is a minor effect (*i-*RBCs *σ*_cell cytoplasm_ = 0.53 ± 0.05 S m^−1^ at 24 hpi). Only after the parasite reaches its later stages, did the ion exchange activity increase. Ions from the high conductivity isotonic medium entered the host cell during the later stages of the life cycle (greater than 24 hpi), causing a sudden increase in *i-*RBCs cytoplasm conductivity between 24 and 42 hpi (*p* < 0.0001). Cytoplasm conductivity increased from *σ*_cell cytoplasm_ = 0.74 ± 0.04 S m^−1^ at 30 hpi, to values close to that of the medium by 42 hpi − *σ*_cell cytoplasm_ =1.24 ± 0.04 S m^−1^. This was much higher than *u-*RBCs (*p* < 0.0001 at 42 hpi), which maintained a constant, much lower value (*c-*RBCs: *σ*_cytoplasm_ = 0.41 ± 0.02 S m^−1^; *u-*RBCs: *σ*_cytoplasm_ = 0.43 ± 0.01 S m^−1^; *N* = 7 time points), in agreement with the published literature [25,51,53,54]. The double-shell model was also used to estimate the cytoplasm conductivity of the parasite itself (figure 7*b*). As with uninfected cells, the parasite maintains a rather stable, lower cytoplasm conductivity along the late stages of TCs: *σ*_par cytoplasm_ = 0.48 ± 0.02 S m^−1^; *N* = 4 time points (the fixed values for early stages are not considered).

Finally, the volumetric changes in the RBC need to be considered. It has been shown that there is no significant alteration in the *i-*RBC volume when compared with *u-*RBC for *P. falciparum* [3]. This is also inferred from the estimated electrical volumes. No statistically significant volume change between *i-*RBCs (94.6 ± 5.14 fl, *N* = 7 time points) and *u-*RBCs (96.3 ± 4.25 fl, *N* = 7 time points) was measured. By contrast, the parasite itself undergoes various volume changes during growth, estimated through the modelling process. During the early stages (≤18 hpi), parasite activity is focused on creating conditions for survival, by consuming haemoglobin, altering the membrane and re-organizing the host cell interior [7,16,59,60]. At this stage, the parasite occupies a very small portion of the host cell, with a volume ratio (i.e. the ratio between the estimated infected cell volume and the estimated parasite volume) of less than 10% at ≤18 hpi (figure 7*c*). As the parasite became metabolically more active (≥24 hpi), it increases in size, reaching a maximum of 90% volume ratio between 36 and 42 hpi (figure 7*c*).

The full dielectric characterization of the *i-*RBCs correlates with the known changes in cell properties over the parasite intraerythrocytic life cycle. Alterations to the host cell dielectric properties, at the membrane and/or cytoplasm level, start from as early as 12 hpi. Exploiting advances in the synchronization of parasites, we have shown significant changes in dielectric properties in different stages of the parasite intraerythrocytic cycle, not previously noted [25]. These differences could be exploited to develop label-free analysis and sorting methods, for example, using AC electrokinetics techniques such as DEP. DEP-based sorting requires two populations of particles to have different dielectric properties (and/or size). Since *i-*RBCs and *u-*RBCs are the same size [3], the differences in dielectric properties could be exploited to identify and sort the *i-*RBCs. The addition of such an enrichment step would make it possible to increase the sensitivity of the ‘gold-standard’ diagnostic method (with a limit of detection of 50 parasites µl^−1^), removing one of the current hurdles to improving the accuracy of diagnosis.

## 4. Conclusion

MIC of *P. falciparum i-*RBCs was used to characterize the time-dependent changes in dielectric properties of the host cell and parasite throughout the 48 h intraerythrocytic life cycle. Parasitized cells were uniquely identified from their fluorescence signature. Impedance analysis was able to differentiate uninfected and *i-*RBCs based on cell membrane properties. The impedance data were modelled using the multi-shell model and while an oblate spheroid model was used for early stages of infection, a spherical model better represents the altered, less discoid *i-*RBC at later stages. Membrane capacitance values for *i-*RBCs increased as the parasite developed (*p* < 0.01 between 6 and 36 hpi) when compared with *u-*RBCs, confirming known host cell membrane alterations. The membrane capacitance of the *i-*RBCs increases from *C*_cell membrane_ = 8.1 ± 0.50 mF m^−2^ at 6 hpi to 13.8 ± 2.47 mF m^−2^ at the end of the parasite intraerythrocytic life cycle (42 hpi). As a consequence of these membrane modifications, the conductivity of *i-*RBCs cytoplasm also changed. There was an increasingly inward flux of ions from the high conductivity, isotonic medium to the lower conductivity interior of the *i-*RBC, notably after 24 hpi when NPPs appear and are more active. The cytoplasmic conductivity of the host cell increased from *σ*_cell cytoplasm_ = 0.53 ± 0.05 S m^−1^ at 24 hpi to 1.24 ± 0.04 S m^−1^ at 42 hpi. It was estimated that the volume ratio occupied by the parasite within the host cell also increased from less than 10%, at earlier stages, to around 90%, at later stages.

These findings could be used to develop new label-free cell sorting techniques for identification and sample pre-enrichment. DEP-based sorting relies on exploiting differences in the dielectric properties of cells, e.g. infected and uninfected cells. This would improve the standard parasite detection protocols, increasing the sensitivity of current diagnostic methods. Although cells cannot be uniquely identified during the ring-stage of the infection cycle, *i-*RBCs could be enriched at other points. While this limits the applicability of a DEP-based diagnosis technique for *P. falciparum i-*RBCs, although not examined in this paper, the technique could also be useful for other *Plasmodium* species where all stages circulate in the peripheral blood.

## Supplementary Material

Electronic Supplementary Material

## Data accessibility

All data supporting this study are openly available from the University of Southampton repository at https://eprints.soton.ac.uk/420483/.
