# Supplementary material for: Dielectric characterization of Plasmodium falciparum-infected red blood cells using microfluidic impedance cytometry
Source: J R Soc Interface. 2018 Oct 17;15(147):20180416. doi: 10.1098/rsif.2018.0416 (PMC6228484; doi:10.1098/rsif.2018.0416)
Supplement: Electronic Supplementary Material [file rsif20180416supp1.docx]

**Dielectric characterization of *Plasmodium falciparum* infected red blood cells using microfluidic impedance cytometry^†^**

C. Honrado^1^, L. Ciuffreda^2^, D. Spencer^1^, L. Ranford-Cartwright^3^ and H. Morgan^1*^

^†^*Electronic Supplementary Information available*

^1^*Faculty of Physical Sciences and Engineering and Institute for Life Sciences, University of Southampton, Southampton, UK, SO17 1BJ*

^2^*Institute of Infection, Immunity and Inflammation, University of Glasgow, Glasgow, UK*

^3^*Institute of Biodiversity, Animal Health and Comparative Medicine, University of Glasgow, Glasgow, UK*

*^*^Corresponding author. Telephone: +442380593330. E-mail: hm@ecs.soton.ac.uk.*

**Electronic Supplementary Material**

1. **Supplementary Figures and Tables**


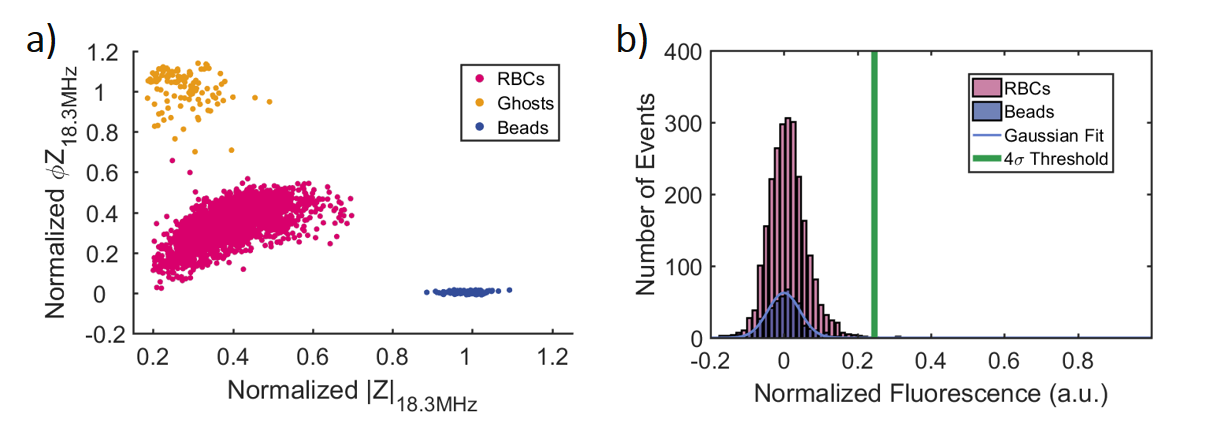


***Figure S1:*** Infected cells identification process applied to control samples. **a)** Normalized impedance scatter plot (magnitude - |Z| versus phase – ΦZ), at reference frequency (18.3 MHz), of a control sample, mixed with reference beads. **b)** Normalized fluorescence distributions of reference beads and RBCs, used for identification of infected cells.


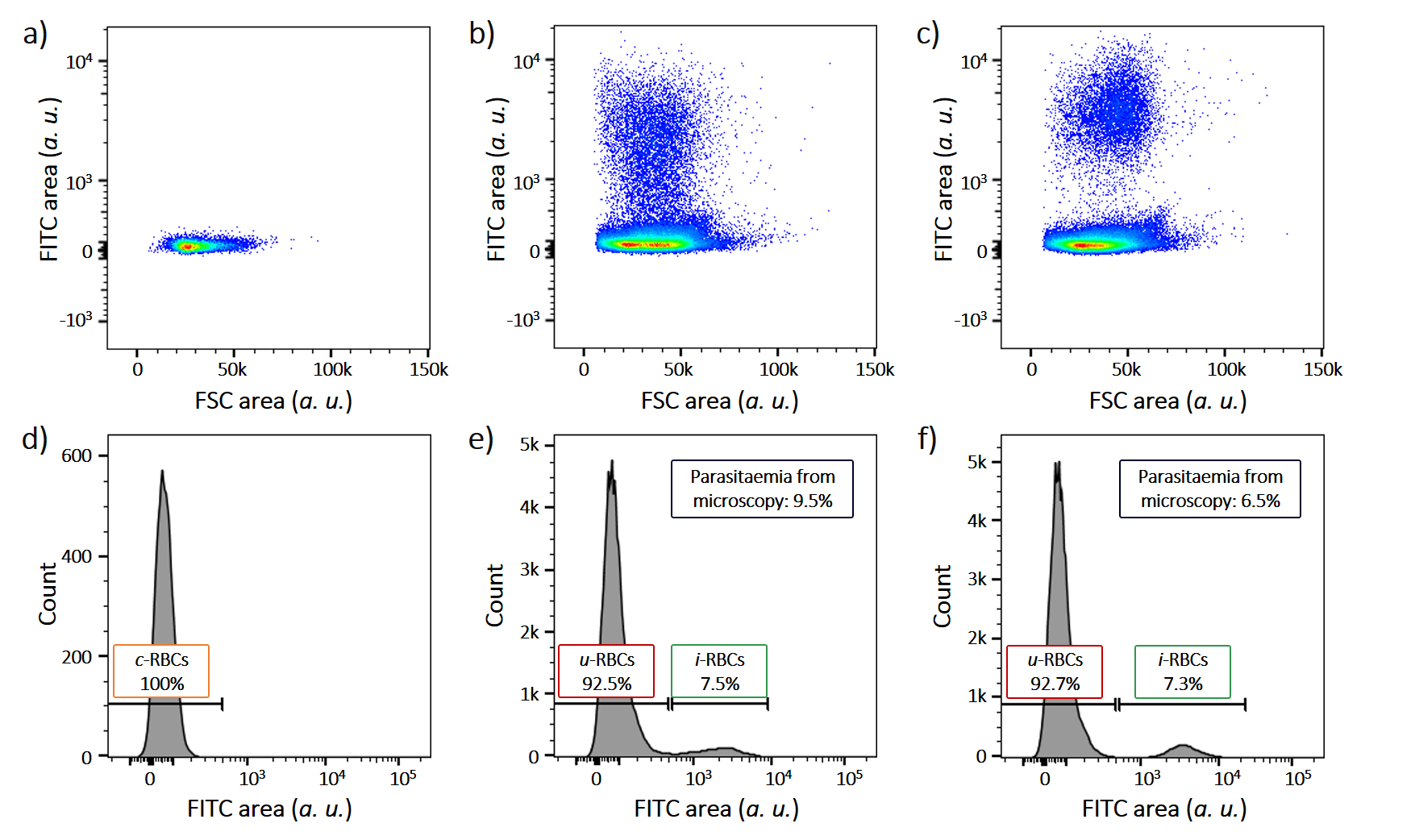


***Figure S2:*** Conventional flow cytometry data for *c-*RBCs (**a** and **d**), early stage *i-*RBCs (**b** and **e**), and late stage *i-*RBCs (**c** and **f**). Forward scatter – FSC is plotted against fluorescence - FITC region (**a**, **b** and **c**), revealing the presence of GFP-parasites in *i-*RBCs populations only. Parasitaemia levels estimated using histograms of fluorescence (**d**, **e** and **f**) are compared with microscopy-based identification (Figure S3).


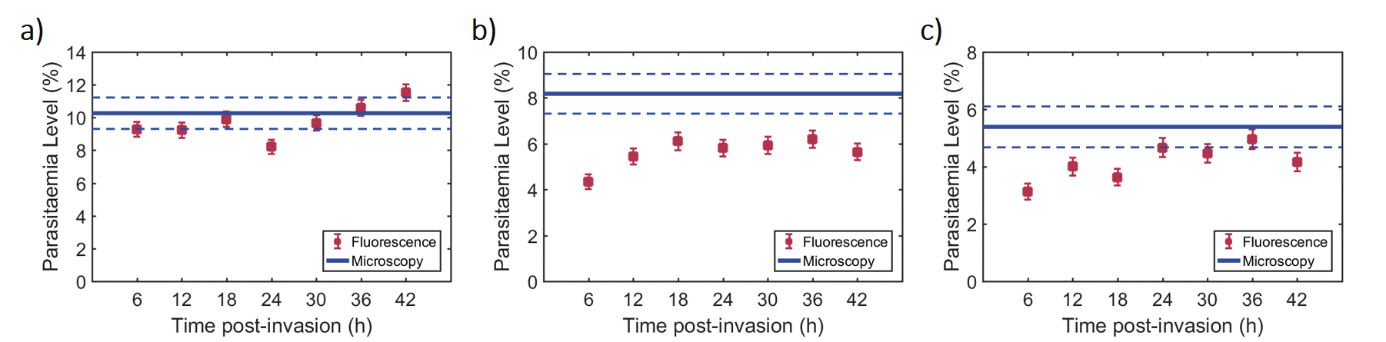


***Figure S3:*** Parasitaemia calculated using Giemsa-staining/ light microscopy- and fluorescence/MIC- based methods for **a)** TC1, **b)** TC2 and **c)** TC3. For Giemsa-staining/ light microscopy, a single mean value (*line*) was calculated for each TC, with a corresponding standard error (*dashed line*) for N=1000 counts. For fluorescence/ MIC, individual values (*squares*) were calculated for each time-point, with corresponding standard error of the mean values (*error bars*) calculated for an average of N=4000 events detected per time-point.

***Table S1:*** Statistical analysis of Giemsa-staining/ light microscopy- versus fluorescence/MIC- based methods for parasitaemia calculations along the time-course of infection (Pearson's chi-squared test for proportions of *i-*RBCs in RBCs populations, N = 7 time-points, n. s. - not significant, *p<0.05, **p<0.01, ***p<0.001 and ****p<0.0001)

| Time-Course | 6 hpi | 12 hpi | 18 hpi | 24 hpi | 30 hpi | 36 hpi | 42 hpi |
| --- | --- | --- | --- | --- | --- | --- | --- |
| 1 | n. s. | n. s. | n. s. | n. s. | n. s. | n. s. | n. s. |
| 2 | ******** | ***** | n. s. | ***** | ***** | ***** | ****** |
| 3 | ****** | n. s. | ***** | n. s. | n. s. | n. s.. | n. s. |

***Table S2:*** Percentage of cells designated as “*i-*RBCs” according to the fluorescence threshold (4× the standard deviation (σ) away from the mean fluorescence of the beads) in control samples *(c*-RBCs) using the fluorescence/MIC- based method.

| Time-Course | Mislabelled “*i-*RBCs” in control samples (%) | | | | | | |  |  | |  |
| --- | --- | --- | --- | --- | --- | --- | --- | --- | --- | --- | --- |
|  | **6 hpi** | **12 hpi** | **18 hpi** | **24 hpi** | **30 hpi** | **36 hpi** | **42 hpi** |  |  | |  |
| 1 | 0.4 | 0.3 | 0.2 | 0.6 | 0.7 | 0.5 | 0.7 |  |  | |  |
| 2 | 0.2 | 0.3 | 0.4 | 0.7 | 0.1 | 0.1 | 0.0 | **Overall Mean** | | **Overall**  **SD** | |
| 3 | 0.3 | 0.0 | 0.2 | 0.1 | 0.3 | 0.0 | 0.1 |  |  |  |  |
| Mean | 0.3 | 0.2 | 0.3 | 0.4 | 0.4 | 0.2 | 0.3 | 0.3 | | 0.1 | |
| SD | 0.1 | 0.1 | 0.1 | 0.3 | 0.2 | 0.2 | 0.3 |  |  |  |  |


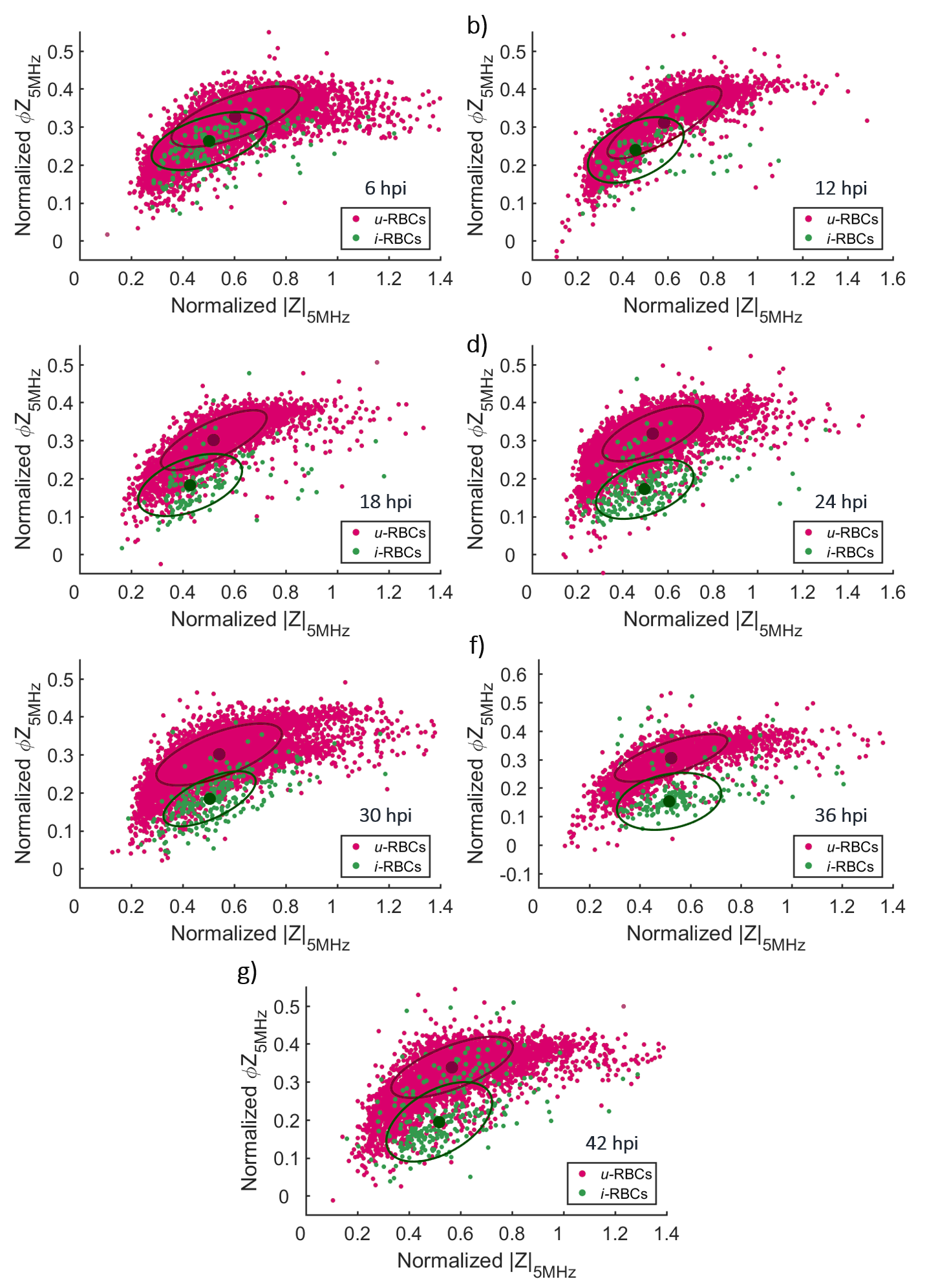


***Figure S4:*** Normalized impedance scatter plots (magnitude - |Z| versus phase – ΦZ), at probe frequency of 5 MHz, of blood samples **a)** 6hpi, **b)** 12 hpi, **c)** 18 hpi, **d)** 24 hpi, **e)** 30hpi, **f)** 36 hpi and **g)** 42 hpi showing discrimination between *u-*RBCs and *i-*RBCs. The mean (●) and ellipse containing 50% of each population are also indicated. Data from TC3.


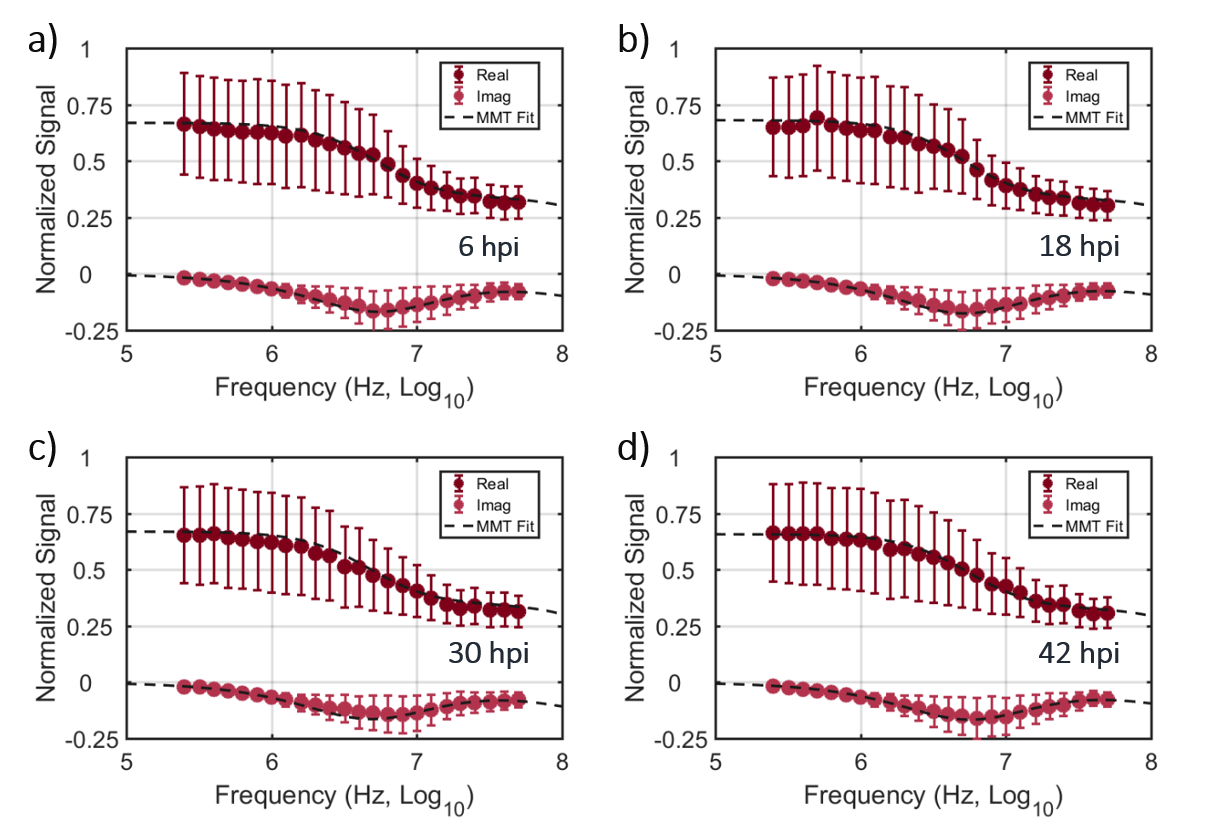


***Figure S5:*** Normalized real and imaginary parts of impedance of RBCs, across the measured probe frequency spectrum, of control blood samples at **a)** 6hpi, **b)** 18 hpi, **c)** 30 hpi and **d)** 42 hpi. Single-shell oblate spheroid models were used to generate each MMT fit. The optimal MMT fits (*dashed lines*) are plotted on top of individual probe frequency mean values (*circles*) and standard deviation (*error bars*) for each time-point. Data from TC2.

***Table S3:*** Dielectric properties along the duration of intraerythrocytic infection, estimated using MMT modelling, of *c-*RBCs

| Properties | Times | | | | | | |
| --- | --- | --- | --- | --- | --- | --- | --- |
|  | **6 hpi** | **12 hpi** | **18 hpi** | **24 hpi** | **30 hpi** | **36 hpi** | **42 hpi** |
| $\boldsymbol{\varepsilon}_{\boldsymbol{membrane}}$ | 4.24 ± 0.21 | 4.38 ± 0.20 | 4.24 ± 0.22 | 4.10 ± 0.31 | 4.20 ± 0.22 | 4.26 ± 0.22 | 4.13 ± 0.36 |
| $\boldsymbol{\varepsilon}_{\boldsymbol{cytoplasm}}$ | 57.8 ± 4.5 | 56.4 ± 1.9 | 58.3 ± 4.2 | 56.9 ± 2.2 | 59.9 ±4.2 | 62.5 ± 6.2 | 54.4 ± 0.7 |
| $\boldsymbol{\sigma}_{\boldsymbol{cytoplasm}}$ (S/m) | 0.38 ± 0.04 | 0.40 ± 0.04 | 0.43 ± 0.01 | 0.40 ± 0.01 | 0.44 ± 0.05 | 0.40 ± 0.04 | 0.44 ± 0.04 |
| $\boldsymbol{C}_{\boldsymbol{membrane}}$ (mF/m^2^) | 7.51 ± 0.36 | 7.76 ± 0.35 | 7.51 ± 0.39 | 7.26 ± 0.55 | 7.45 ± 0.40 | 7.54 ± 0.39 | 7.32 ± 0.64 |


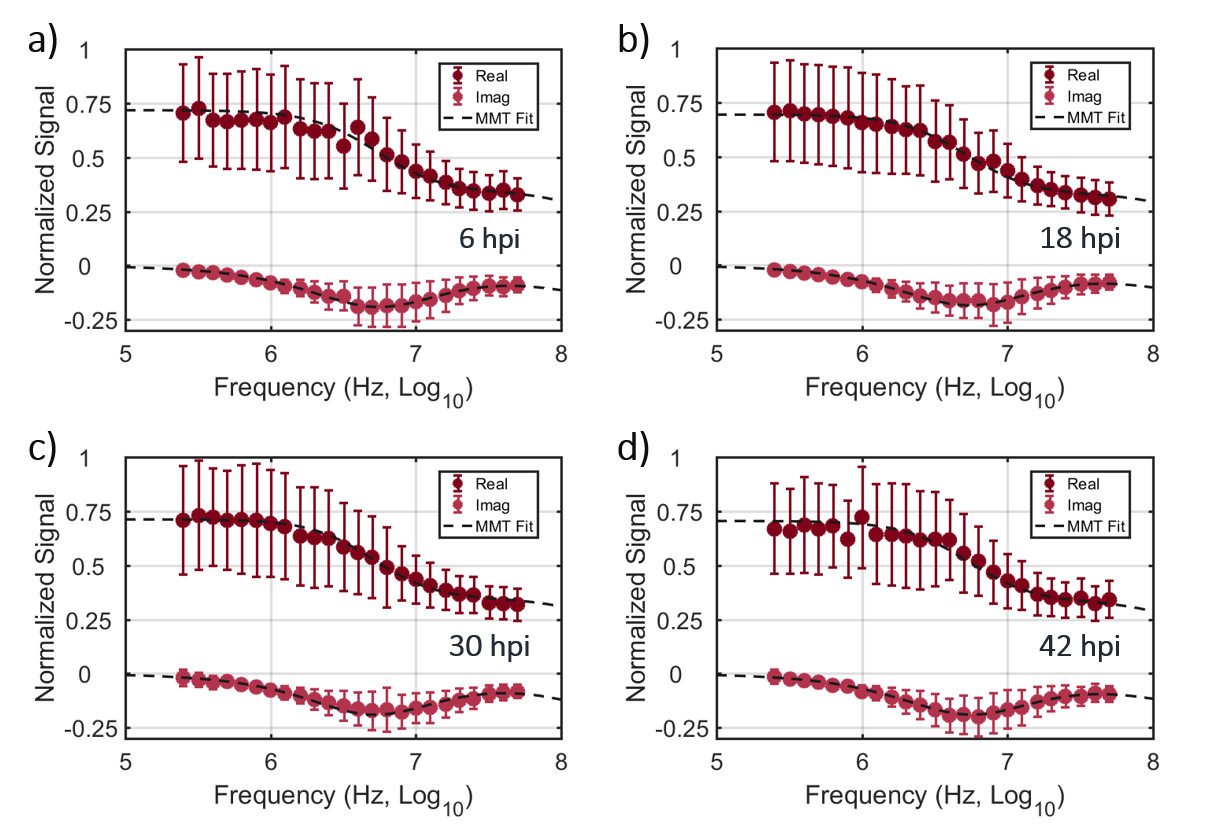


***Figure S6:*** Normalized real and imaginary parts of impedance of *u-*RBCs, across the measured probe frequency spectrum, from cultures at **a)** 6hpi, **b)** 18 hpi, **c)** 30 hpi and **d)** 42 hpi. Single-shell oblate spheroid models were used to generate each MMT fit. The optimal MMT fits (*dashed lines*) are plotted on top of individual probe frequency mean values (*circles*) and standard deviation (*error bars*) for each time-point. Data from TC3.

***Table S4:*** Dielectric properties along the duration of intraerythrocytic infection, estimated using MMT modelling, of *u-*RBCs

| Properties | Times | | | | | | |
| --- | --- | --- | --- | --- | --- | --- | --- |
|  | **6 hpi** | **12 hpi** | **18 hpi** | **24 hpi** | **30 hpi** | **36 hpi** | **42 hpi** |
| $\boldsymbol{\varepsilon}_{\boldsymbol{membrane}}$ | 4.01 ± 0.05 | 3.95 ± 0.05 | 4.07 ± 0.04 | 4.04 ± 0.17 | 4.10 ± 0.28 | 3.93 ± 0.14 | 4.00 ± 0.05 |
| $\boldsymbol{\varepsilon}_{\boldsymbol{cytoplasm}}$ | 53.7 ± 5.1 | 59.1 ± 4.8 | 60.5 ± 5.0 | 59.2 ± 5.6 | 56.0 ±0.8 | 64.1 ± 2.2 | 60.3 ± 3.1 |
| $\boldsymbol{\sigma}_{\boldsymbol{cytoplasm}}$ (S/m) | 0.41 ± 0.02 | 0.45 ± 0.02 | 0.44 ± 0.03 | 0.43 ± 0.02 | 0.41 ± 0.01 | 0.43 ± 0.01 | 0.43 ± 0.02 |
| $\boldsymbol{C}_{\boldsymbol{membrane}}$ (mF/m^2^) | 7.10 ± 0.10 | 7.00 ± 0.09 | 7.20 ± 0.07 | 7.15 ± 0.30 | 7.27 ± 0.49 | 6.96 ± 0.25 | 7.09 ± 0.08 |


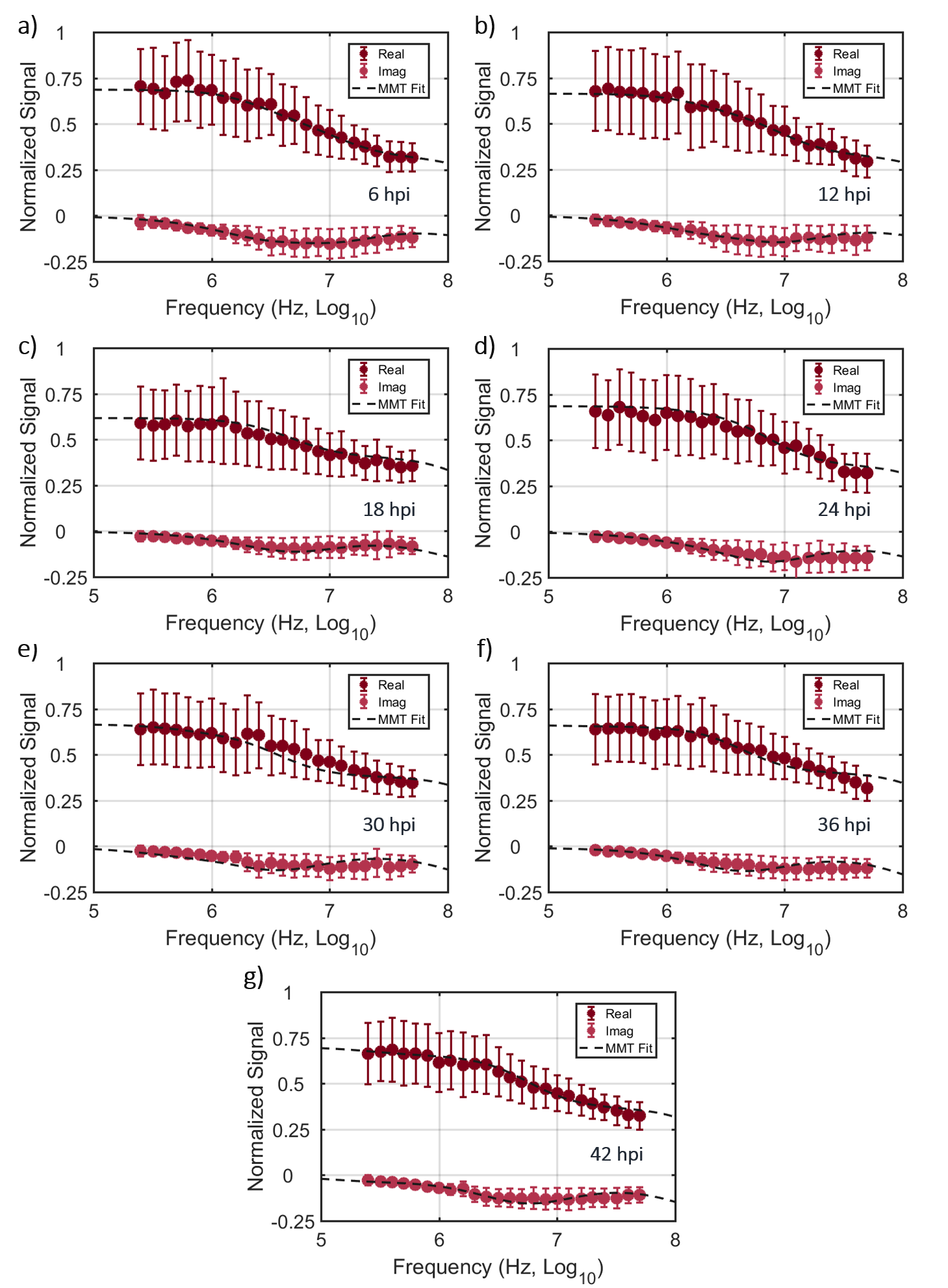


***Figure S7:*** Normalized real and imaginary parts of impedance of *i-*RBCs, across the measured probe frequency spectrum, of cultures at **a)** 6hpi, **b)** 12 hpi, **c)** 18 hpi, **d)** 24 hpi, **e)** 30hpi, **f)** 36 hpi and **g)** 42 hpi. Double-shell oblate spheroid (**a**, **b** and **c**) and spherical (**d**, **e**, **f** and **g**) models were used to generate each MMT fit. The optimal MMT fits (*dashed lines*) are plotted on top of individual probe frequency mean values (*circles*) and standard deviation (*error bars*) for each time-point. Data from TC1.

***Table S5:*** Statistical analysis of host cell membrane capacitance changes in *i-*RBCs along the time-course of infection (Tukey’s test, N = 21 pairwise comparisons; n. s. - not significant, *p<0.05, **p<0.01)

| $\boldsymbol{C}_{\boldsymbol{cell membrane}}$ | 6 hpi | 12 hpi | 18 hpi | 24 hpi | 30 hpi | 36 hpi | 42 hpi |
| --- | --- | --- | --- | --- | --- | --- | --- |
| 6 hpi |  | n. s. | n. s. | ***** | ****** | ****** | ****** |
| 12 hpi |  |  | n. s. | n. s. | ***** | ****** | ***** |
| 18 hpi |  |  |  | n. s. | n. s. | n. s. | n. s. |
| 24 hpi |  |  |  |  | n. s. | n. s. | n. s. |
| 30 hpi |  |  |  |  |  | n. s. | n. s. |
| 36 hpi |  |  |  |  |  |  | n. s. |
| 42 hpi |  |  |  |  |  |  |  |

***Table S6:*** Statistical analysis of host cell cytoplasmic conductivity changes in *i-*RBCs along the time-course of infection (Tukey’s test, N = 21 pairwise comparisons; n. s. - not significant, *p<0.05, **p<0.01, ***p<0.001 and ****p<0.0001)

| $\boldsymbol{\sigma}_{\boldsymbol{cell cytoplasm}}$ | 6 hpi | 12 hpi | 18 hpi | 24 hpi | 30 hpi | 36 hpi | 42 hpi |
| --- | --- | --- | --- | --- | --- | --- | --- |
| 6 hpi |  | n. s. | n. s. | n. s. | ****** | ****** | ******** |
| 12 hpi |  |  | n. s. | n. s. | ****** | ****** | ******** |
| 18 hpi |  |  |  | n. s. | ****** | ****** | ******** |
| 24 hpi |  |  |  |  | ****** | ****** | ******** |
| 30 hpi |  |  |  |  |  | ***** | ******* |
| 36 hpi |  |  |  |  |  |  | ***** |
| 42 hpi |  |  |  |  |  |  |  |

1. **Theory**

A cell is typically modelled as a conducting particle (cell interior) surrounded by an insulating cell membrane, suspended in a conducting medium; its dielectric properties are determined using the so-called multi-shell model, which describes the cell as a series of concentric shells with defined dielectric properties (1–5). Maxwell’s mixture theory (MMT) (6) is used to correlate the properties of the measured particle to its intrinsic properties (6,7). The single-shell model is the simplest model that describes the dielectric properties of a cell that exhibits a single relaxation due to the presence of the cell membrane. Figure S8a shows how the different component parts of the cell modify the frequency-dependent electrical response. The presence of the lipid cell membrane means that cells behave as insulating particles at low frequencies (kHz). However at higher frequencies (MHz), the cell appears progressively more conductive as the electric field probes intracellular properties (5,7,8). In the absence of any substantive membrane conductivity, the cell volume dominates the response at low frequencies (<1 MHz), while at intermediate frequencies (1-10 MHz) the membrane capacitance dominates (9,10). Cytoplasm properties are measured at still higher frequencies (>10MHz), when the electric field capacitively couples across the membrane and the impedance signal reflects the cell interior.

For a particle exhibiting two dielectric relaxations the double-shell model provides the best approximation - Figure S8b. For higher frequencies (>10 MHz), after the relaxation of the outer membrane, the impedance signal is a function of the cell cytoplasmic properties. As frequency increases, impedance eventually measures the properties of the second (internal) shell *i.e.*, the dielectric properties of the parasite, with the second dielectric relaxation occurring at much higher frequencies (7). The dielectric properties of both host cell and parasite can thus be approximated based on these dielectric relaxations.


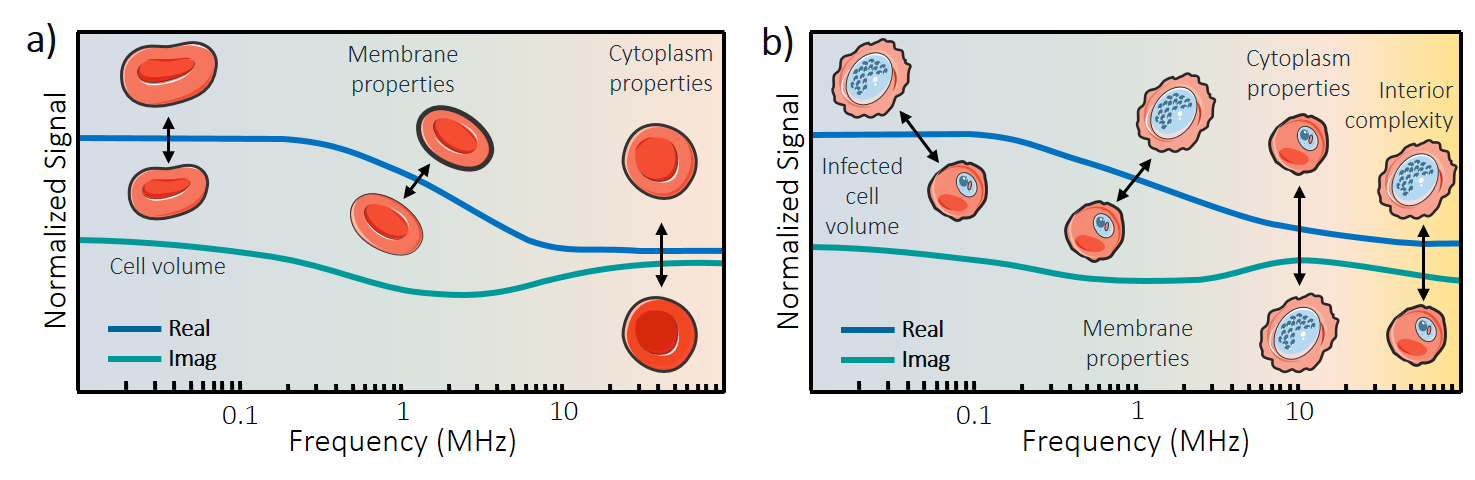


**Figure S8:** Illustration of the frequency-dependent dielectric response of **a)** a single-shelled particle (as u-RBCs), with a single relaxation, and **b)** a double-shelled particle (as i-RBCs), with two relaxations. The relationships between some of the dielectric properties and behaviour at specific frequencies are highlighted. Note that the frequency window for these relaxations depends on the conductivity of the suspending medium.

A more suitable model for discoid shaped RBCs is the ellipsoidal model, as represented in Figure 5, for an oblate spheroid (7,8,11–14). Consider a homogeneous, ellipsoidal, dielectric particle, with random orientation, and semi-axes $n$ = $a,b$ and $c$. The effective polarizability for each semi-axis ($\tilde{\alpha}_{n}$) is different and can be calculated from:

$\tilde{\alpha}_{n}=3 \tilde{\varepsilon}_{medium} \tilde{K}_{n}$ (1)

where $\tilde{\varepsilon}_{medium}$ is the complex permittivity of the suspending medium. The polarizability, and thus the effective dipole moment of the particle, is frequency dependent and described by the factor:

$\tilde{K}_{n}= \frac{\tilde{\varepsilon}_{particle}-\tilde{\varepsilon}_{medium}}{3(A_{n}\left( \tilde{\varepsilon}_{particle}-\tilde{\varepsilon}_{medium} \right)+\tilde{\varepsilon}_{medium})}$ (2)

This factor is different for each semi-axis *n*, and depends on a depolarising factor $A_{n}$. For the case of an oblate spheroid ($a<b=c$), such as RBCs, $A_{n}$ takes the form:

$A_{a}=\frac{1}{1-q^{2}}+\frac{q}{\left( 1-q^{2} \right)^{\frac{3}{2}}}arcos(q)$ (3)

$A_{b}=A_{c}=(1-A_{a})/2$

where $q$ is the ratio of semi-axis ($a/b$). For the case of spherical particles ($a=b=c$), such as late stage *i-*RBCs, the depolarising factors are simplified to $A_{a}=A_{b}=A_{c}=1/3$.

The relaxation in the effective polarizability of a particle is described by the Clausius-Mossotti factor ($\tilde{f}_{CM}$). It can be approximated taking in consideration the polarizability along each semi-axis:

$f_{CM}=A_{a} \tilde{K}_{a}+A_{b} \tilde{K}_{b}+A_{c} \tilde{K}_{c}$ (4)

For a particle in a suspending medium at low volume fraction ($\varphi\ll$ 1), Maxwell’s mixture theory gives the equivalent complex dielectric permittivity of that mixture as:

$\tilde{\varepsilon}_{mix}=\tilde{\varepsilon}_{medium}\frac{1+2\varphi\tilde{f}_{CM}}{1-\varphi\tilde{f}_{CM}}$ (5)

This equation gives the dielectric decrement due to the presence of the particle in the suspending medium between the measurement electrodes. The impedance of the mixture ($\tilde{Z}_{mix}$) can be calculated:

$\tilde{Z}_{mix}=\frac{1}{i\omega\tilde{\varepsilon}_{mix}G_{f}}$ (6)

where $i^{2}=-1$, $\omega$ is the angular frequency, and $G_{f}$ is the geometric constant of the system. For an ideal parallel plate electrode system, $G_{f}$ is simplified to ${A_{electrode}}/{d_{electrode}}$, where $A_{electrode}$ is the electrode surface area and $d_{electrode}$ is the separation distance between electrodes. For non-ideal systems, the electric field is no longer uniform in the entire volume and the effect of non-uniform field has to be taken into account (15–18). The Real ($\mathfrak{R}\left( \tilde{Z}_{mix} \right)$) and Imaginary ($\mathfrak{I}(\tilde{Z}_{mix})$) parts of the mixture impedance were calculated, and the corresponding relaxation curves generated. By fitting each curve to the Real and Imaginary parts of the experimental impedance data, the dielectric properties of the particle can be estimated.

**References**

1. Hanai T, Koizumi N, Irimajiri A. A method for determining the dielectric constant and the conductivity of membrane-bounded particles of biological relevance. Biophys Struct Mech. 1975;1(4):285–94.

2. Irimajiri A, Hanai T, Inouye A. A dielectric theory of “multi-stratified shell” model with its application to a lymphoma cell. J Theor Biol. 1979;78(2):251–69.

3. Huang Y, Hölzel R, Pethig R, Wang XB. Differences in the AC electrodynamics of viable and non-viable yeast cells determined through combined dielectrophoresis and electrorotation studies. Phys Med Biol. 1992;37(7):1499–517.

4. Wang XB, Huang Y, Gascoyne PRC, Becker FF, Hölzel R, Pethig R. Changes in Friend murine erythroleukaemia cell membranes during induced differentiation determined by electrorotation. BBA - Biomembr. 1994;1193(2):330–44.

5. Schwan HP. Advances in Biological and Medical Physics. 5th ed. Tobias CA, Lawrence JH, editors. New York: Academic Press Inc.; 1957. 147 p.

6. Maxwell JC. A treatise on electricity and magnetism [Internet]. Vol. 1, A treatise on electricity and magnetism. Clarendon Press; 1881. Available from: http://ebooks.cambridge.org/ref/id/CBO9781107415324A009

7. Morgan H, Green NG. AC Electrokinetics: colloids and nanoparticles. Baldock, Hertfordshire, UK: Research Studies Press Ltd.; 2002.

8. Raicu V, Feldman Y. Dielectric Relaxation in Biological Systems. Oxford, Oxfordshire, UK: Oxford University Press; 2015. 464 p.

9. Ashrafuzzaman M, Tuszynski J. Membrane Biophysics. Springer; 2012.

10. Gascoyne P, Shim S, Noshari J, Becker FF, Stemke-Hale K. Correlations between the Dielectric Properties and Exterior Morphology of Cells Revealed by Dielectrophoretic Field-Flow Fractionation. Electrophoresis. 2013;34(7):1–20.

11. Gimsa J, Müller T, Schnelle T, Fuhr G. Dielectric spectroscopy of single human erythrocytes at physiological ionic strength: dispersion of the cytoplasm. Biophys J [Internet]. 1996;71(1):495–506. Available from: http://www.pubmedcentral.nih.gov/articlerender.fcgi?artid=1233500&tool=pmcentrez&rendertype=abstract

12. Asami K, Yonezawa T. Dielectric behavior of non-spherical cells in culture. BBA - Gen Subj. 1995;1245(3):317–24.

13. Miller RD, Jones TB. Electro-orientation of ellipsoidal erythrocytes. Theory and experiment. Biophys J [Internet]. 1993;64(5):1588–95. Available from: http://eutils.ncbi.nlm.nih.gov/entrez/eutils/elink.fcgi?dbfrom=pubmed&id=8324193&retmode=ref&cmd=prlinks

14. Asami K. Dielectric approach to suspensions of ellipsoidal particles. Jpn J Appl Phys. 1980;19(2):359–65.

15. Sun T, Bernabini C, Morgan H. Single-colloidal particle impedance spectroscopy: Complete equivalent circuit analysis of polyelectrolyte microcapsules. Langmuir. 2010;26(6):3821–8.

16. Sun T, Morgan H. Single-cell microfluidic Impedance cytometry: A review. Microfluid Nanofluidics. 2010;8(4):423–43.

17. Wachner D, Simeonova M, Gimsa J. Estimating the subcellular absorption of electric field energy: Equations for an ellipsoidal single shell model. Bioelectrochemistry. 2002;56(1–2):211–3.

18. Sun T, Green NG, Gawad S, Morgan H. Analytical electric field and sensitivity analysis for two microfluidic impedance cytometer designs. IET nanobiotechnology. 2007;1(5):69–79.
